# Genetic strategies for avian influenza resilience in poultry: from host-pathogen interaction studies to precision breeding

**DOI:** 10.1007/s10142-025-01777-w

**Published:** 2025-11-24

**Authors:** Debolina Majumdar, Emily Hann, Kirsty R. Short, Karel A. Schat, Arjun Challagulla

**Affiliations:** 1 Australian Centre for Disease Preparedness, CSIRO Health and Biosecurity, Geelong, 3220 Australia; 2 School of Life and Environmental Sciences, Deakin University, Geelong, VIC Australia; 3 School of Chemistry and Molecular Biosciences, The University of Queensland, St Lucia, QLD 4072 Australia; 4 Australian Infectious Diseases Research Centre, The University of Queensland, St Lucia, QLD 4072 Australia; 5 Department of Microbiology and Immunology, College of Veterinary Medicine, Cornell University, Ithaca, NY 14853 USA

**Keywords:** Influenza virus, Genome editing, Genome-wide screening, Poultry, Disease resilience

## Abstract

Avian influenza virus (AIV) remains a persistent threat to global poultry production and public health, owing to its ability to infect a wide range of avian and mammalian species. The global resurgence of H5N1 and the limitations associated with control measures in intensive poultry production have highlighted the need for a paradigm shift in disease management strategies. In this context, the development of AIV resilient poultry lines is gaining momentum as a promising strategy for long-term disease management. In this review, we discuss the ongoing threat of AIV to poultry production and explore genetic approaches to enhance resilience against avian influenza. We primarily focus on intracellular host restriction factors that inhibit viral replication, followed by an overview of virus-targeted strategies. We further discuss the use of genome-wide screening approaches to study host–pathogen interactions to identify high confidence host targets. Finally, we highlight studies that develop transgenic, or genome engineered chickens with the aim to enhance AIV resilience and their potential for transforming AIV control in poultry production.

## Introduction

The Food and Agriculture Organization (FAO) estimates that a 70% increase in food production is necessary to feed the 9.7 billion population expected by 2050 (reviewed in FAO 2009). Furthermore, rising income levels and increased affordability are influencing consumer preferences for protein-rich and nutritionally balanced diets. Poultry products, including chicken meat and eggs, have become the primary source of affordable, high-quality animal-derived proteins globally. Estimates from the FAO indicate that poultry meat will constitute approximately 40% of global meat consumption by 2032. This growing demand for poultry products underscores the critical role that poultry will play in future food security (reviewed in (OECD/FAO 2023). Poultry production has a long-standing history of implementing innovative strategies, with a specific emphasis on genetic improvements and the selection of breeds that exhibit enhanced agronomic and production traits. Such innovations have led to improvements in growth rate, reproductive performance, feed conversion yield, and overall production efficiency (reviewed in Siegel 2014; Neeteson et al. 2023). For example, typical commercial broiler chickens attain market weight within 6 weeks, whereas commercial laying hens may produce approximately 340 to 480 eggs/hen at 80 and 100 weeks of age, respectively (Hy-Line International n.d.). Despite these advancements, poultry production continues to face challenges in disease management, those arising from protozoa such as Eimeria species, bacterial and viral pathogens. Among the latter, avian influenza virus (AIV) remains a persistent threat to poultry production especially with the current highly pathogenic (hp)AIV H5N1 2.3.4.4b clade causing a world-wide pandemic (Bellido-Martin et al. 2025). The widespread outbreaks cause substantial production and economic losses, as well as the risk of zoonotic transmission. This review summarises the escalating threat posed by AIV to poultry production and ongoing research efforts to identify and implement genetic strategies aimed at enhancing disease resistance in poultry.

## Avian influenza virus

Influenza A virus (IAV) is the etiological agent of influenza, a highly infectious respiratory disease that affects a wide range of avian and mammalian species. IAVs belong to the Orthomyxoviridae family, which are enveloped RNA viruses with segmented genomes. The genome of IAV comprises of eight single-stranded, negative-sense RNA segments that encode at least ten proteins. IAV are classified based on the genetic and antigenic characteristics of the surface antigens hemagglutinin (HA) and neuraminidase (NA), of which 18 HA and 11 NA proteins have been isolated from diverse animal species. IAVs that are capable of infecting avian hosts are referred to as avian influenza viruses (avian IAV), which predominantly belong to the H5, H7 and H9 subtypes (reviewed in Nuñez and Ross 2019; Dabrera 2024; Luczo and Spackman 2025; Tan et al. 2025).

The natural reservoir host species of AIV are wild water birds and shorebirds belonging to the orders Anseriformes and Charadriiformes. In these bird species, AIVs circulate endemically, maintaining extensive genetic diversity and occasionally spilling over into susceptible species, where they can cause disease. Based on their pathogenicity in chickens, AIVs are further categorized into low pathogenic avian influenza (LPAI) and highly pathogenic avian influenza (HPAI) (reviewed in Taubenberger and Morens 2008; Blagodatski et al. 2021). The proteolytic cleavage of the viral HA precursor (HA0) by cellular proteases determines viral tropism and disease severity in susceptible species. LPAI viruses harbour a monobasic motif (PQ_RETR/G) in HA0 that can be cleaved by trypsin-like serine proteases, which are predominantly present in the respiratory and gastrointestinal tracts, thus restricting viral tropism to these tissues. In contrast, HPAIV contains a polybasic motif (PQ_RERRRKKR/G) that enables HA0 cleavage by widely distributed subtilisin-like proteases, such as furin, facilitating viral replication in diverse tissues and promoting systemic infection (Bertram et al. 2010; Zhang et al. 2021). The propensity for vascular endothelial cell infection in chickens, coupled with the ability to induce systemic infection, is a critical factor for fulminant disease and high mortality rates observed in this species (reviewed in Looi et al. 2018; de Bruin et al. 2022).

### Impact of H5N1 on poultry production

Since its first detection in 1996, the A/Goose/Guangdong/96 (Gs/GD) H5N1 HPAIV has undergone extensive genetic diversification and emerged into several phylogenetic clades (Wan 2012). By 2013, clade 2.3.4.4 of the Gs/GD H5N1 strain had become endemic in China and subsequently disseminated across Asia and beyond, which led to the emergence of novel H5Nx strains, including H5N2, H5N5, H5N6, and H5N8. This intense circulation of H5NX led to the emergence of a panzootic 2.3.4.4b H5N1 virus (reviewed in Wille and Barr 2022), which possessed increased ability to overcome species barriers compared to H5Nx strains that have emerged over the past two decades. The multi-host ecology of clade 2.3.4.4b has caused epizootics across Europe, Africa, Asia, the Americas, and Antarctica, resulting in substantial losses of both avian (wild and domestic) and mammalian marine and terrestrial populations including cattle in the USA (Islam et al. 2023; Kandeil et al. 2023; Caserta et al. 2024). Furthermore, the continued circulation of H5N1 2.3.4.4b in mammalian species have enabled the adaptation for airborne transmission. Mammal to mammal transmission was reported in marine animals and farmed minks, and more recently in dairy cattle (Pardo-Roa et al. 2025).

The impact of H5N1 2.3.4.4 on commercial poultry production is profound, with several millions of poultry being culled since 2020 (reviewed in Ciminski et al. 2023; Klaassen and Wille 2023). Given that modern poultry production involves rearing of birds at high densities and in confined spaces, incursion of influenza viruses greatly influences the evolution and transmission dynamics, causing catastrophic effects on the flocks. To this end, controlling the disease in intensively farmed species such as poultry is critical for mitigating the broader impact of H5N1 on livestock production and human health. Although vaccination of poultry is considered as a potential control strategy, the extensive diversity of field strains presents a major challenge for their effectiveness (Li et al. 2025). This is further compounded by logistical challenges, particularly in broiler production, in which birds reach market weight within 5–7 wk. Furthermore, based on commercial considerations vaccination in poultry has not yet been implemented in several regions of the world, including the USA. To this end, current mitigation strategies to mitigate HPAIV in poultry production continues to rely heavily on stringent biosecurity measures and the culling of infected flocks.

## Genetic approaches for avian influenza resilience

Increasing resilience of chickens to AIV infection will be a valuable trait for intensive poultry production with the potential to transform disease management practices in combination with vaccination and increased biosecurity. Such a combined approach may reduce the need to cull infected flocks, which is costly and may not be effective. Although, vaccine-induced immunity might play a crucial role in controlling AIV outbreaks, it may not prevent superinfection thus contributing to the selection of escape mutants. In contrast, genetic approaches aimed at enhancing resilience traits offer a more sustainable solution because these traits can be inherited across generations within established poultry breeding programs. This is particularly relevant to the poultry production as the genetics of most commercial breeds are maintained by a limited number of breeding companies (reviewed in Siegel 2014; Neeteson et al. 2023). Although early research was mostly focused on identifying key quantitative trait loci (QTL) or genes associated with AIV resistance, the polygenic nature of resilient traits often poses challenges in identifying high-confidence disease-resistance alleles or QTLs (Abasht et al. 2006; Cheng et al. 2013). Furthermore, selective breeding for disease resistance presents a complex and multifaceted challenge because of the difficulties associated with accurately predicting genetic gains between resistant and susceptible chickens. These challenges underscore the need for innovative strategies to overcome the current limitations.

One promising approach to overcome these limitations is by implementation of precision breeding for producing resistant or resilient chicken lines, an approach that presents a perfect complement for selective breeding. The approval of AquaAdvantage salmon and porcine reproductive and respiratory syndrome (PRRS) resistant pigs by the USA Food and Drug Administration (FDA) for human consumption (Green 2016; Burger et al. 2024) underscores the changing regulatory landscape of genetically modified animals for human consumption. Precision breeding of resilient chicken lines using genome editing could accelerate the development of avian influenza resilient chickens by alleviating the shortcomings associated with traditional selective breeding. However, such an implementation depends largely on identifying high confidence host factors or antiviral transgenes that effectively block viral replication. To this end, genome-wide functional screening platforms that enable a deeper understanding of virus-host interactions in an unbiased and systematic manner can accelerate these efforts. Furthermore, programmable nucleases or proteins with highly specific antiviral properties have been harnessed for direct targeting of viral RNA or proteins within host cells and are widely adopted. The latter approach requires the transgenic expression of antiviral effectors, such as small hairpin RNAs (shRNAs), synthetic decoy RNAs, or viral restriction factors. The following sections highlight antiviral host factors identified through studies of host-pathogen interactions and evaluate their potential application in disease-resistant chickens.

### Viral antagonisms identified from host-pathogen interaction studies

The replication cycle of IAV begins with the attachment of viral HA molecules to sialic acid residues on the host cell membrane, with avian strains typically exhibiting a preference for α-(2,3) linkages and human strains favouring α-(2,6) linkages. The attachment of IAV to cell membrane triggers endocytosis and subsequent release of the viral ribonucleoproteins (vRNPs) into the host cytoplasm for genome replication (reviewed in Samji 2009; Zhao and Pu 2022; Du et al. 2023). At each stage of the viral replication cycle, influenza viruses undergo a constant evolutionary arms race with the host immune system. Numerous host-pathogen interaction studies have elucidated the fundamental mechanisms by which influenza viruses exploit host cellular machinery while also identifying cell-intrinsic viral restriction factors. One of the key features of these host viral restriction factors is their ability to inhibit viral replication, either by directly targeting viral genes or proteins, or by activating immune responses by establishing an antiviral state.

**Fig. 1:**
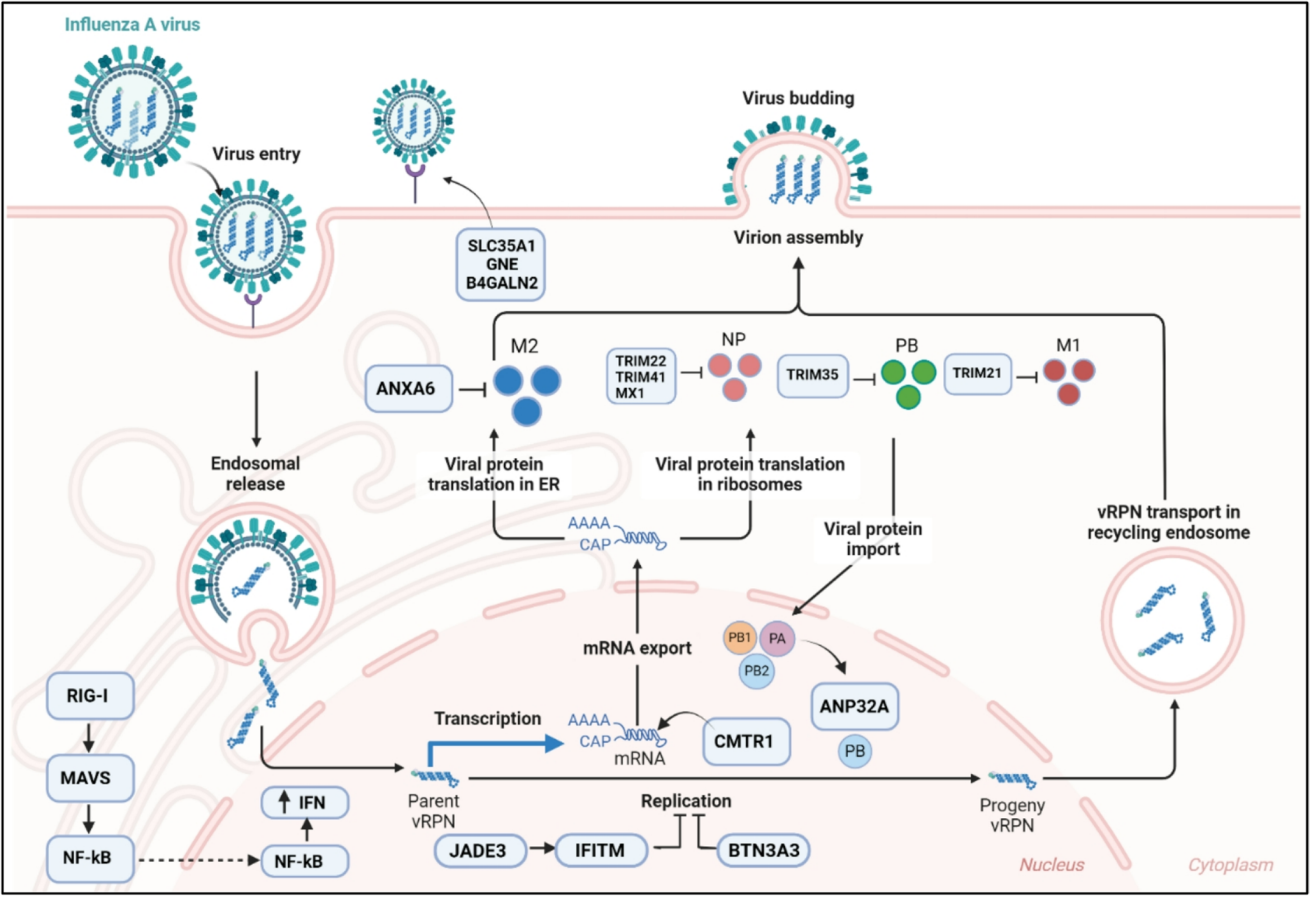
Host-virus interactions highlighting critical host genes in influenza virus infection. This schematic representation depicts the interactions between influenza virus and various host genes critical for influenza infection. Adapted from “Influenza virus life cycle”, by BioRender.com (2024). Retrieved from https://app.biorender.com/biorender-templates

The innate immune system acts as the primary defence mechanism by restricting viral replication, inhibiting the spread of infection, and facilitate adaptive immunity for viral clearance. The type I interferon (IFN) response constitutes a potent and universal intracellular defence mechanism against viral invasion of animal cells. Conventionally, an increased type I IFN response is often linked to enhanced resistance to viral infections in the host. This Type I IFN response begins with the recognition of viral RNA through the extensive repertoire of pattern recognition receptors (PRRs), such as Retinoic acid-inducible gene I (RIG-I) like receptors (RLRs), Toll-like receptors (TLRs), nucleotide-binding oligomerization domain (NOD)-like receptors (NLRs). Activation of these innate immune specific receptors induces a multiprotein signalling complex and a cascade of signal transduction events, culminating in the secretion of type I IFNs, pro-inflammatory cytokines, and chemokines. Finally, these IFNs bind to the cell receptors of adjacent cells and activate the Janus kinase-signal transducer and activator of transcription (JAK-STAT) pathway, which results in the transcriptional regulation of numerous genes collectively termed IFN stimulated genes (ISGs) to establish an antiviral state (reviewed in Kawai and Akira 2006; Raftery and Stevenson 2017). Host factors that restrict viral replication can be broadly categorized into two groups: genes involved in active innate immunity, such as ISGs, and those with inherent antiviral activity that are independent of IFNs (non-ISGs). For example, key ISGs such as the Mx, RIG-I, IFTIMs, TRIM proteins and BTN3A3 have been well studied for their antiviral properties against influenza virus infection. Such findings provide valuable insights into the antiviral potential of naturally occurring genes and elucidate a pathway to enhance AIV resilience in chickens. The following sections will discuss key host genes involved in influenza pathogenesis and previously identified candidate factors implicated in reducing viral replication. Target genes that are amenable for host-directed strategies have been highlighted (Fig. 1).

#### Mx

Mx proteins are members of the dynamin-like GTPase superfamily that are important in a wide range of eukaryotic cellular processes, including the antiviral mechanism by regulating type I (α and β) and type III (λ) IFNs in virus-infected cells (reviewed in Verhelst et al. 2013; Haller et al. 2018). Since its discovery almost 50 years ago, Mx-mediated intracellular defence mechanism in vertebrate species has been studied against a broad spectrum of viruses, including influenza virus. Mx protein comprises a N-terminal GTPase domain, a middle domain and C-terminal GTPase effector domain. The GTPase domain of Mx proteins is essential for antiviral activity, in which the tripartite GTP-binding motif within the GTPase domain facilitates GTP binding and hydrolysis. Phylogenetic analysis of Mx genes indicated that Mx genes have evolved into multiple paralogs and orthologs, each exhibiting distinct subcellular localization and anti-influenza activity across a diverse array of animal species. For example, mouse Mx1(Mu Mx1) is predominantly localised in the nucleus inhibiting influenza viral RNA (vRNA) transcription. In contrast, human Mx1 (also known as MxA) is primarily localised in the cytoplasm and interfere with the vRNP trafficking (Briggs et al. 2024). In avian species, including chickens and ducks, a single Mx gene has been identified (reviewed in Verhelst et al. 2013). The chicken Mx protein is predominantly localized in the cytoplasm and lacks antiviral activity against AIV. Given that influenza virus transcription and replication occur in the nucleus, it was reasonable to hypothesize that redirecting chicken Mx to the nucleus through artificial nuclear localization signals may confer antiviral activity. However, despite successfully localising in the nucleus, chicken Mx failed to exhibit antiviral activity against AIV, suggesting that its cytoplasmic localization is not the sole determinant of resistance and that additional factors may be involved. Moreover, chicken Mx is polymorphic, with a single nucleotide polymorphism at residue 631, which has been studied extensively for its role in restricting AIV replication in chickens. It was originally hypothesized that serine (631Ser) is associated with susceptibility while asparagine (631Asn) confers resistance (Ko et al. 2002). However, follow-up investigations rejected the hypothesis of the potential importance of the S631N polymorphism in Mx-induced influenza restriction (Benfield et al. 2010). In a landmark study, Schusser et al. (2011) demonstrated that chicken Mx lacks the GTPase activity which is a critical component to exert antiviral activity against IAV (Pitossi et al. 1993). This study also demonstrated that protection against influenza infection is independent of Mx-631Asn or Mx-631Ser, and that residue 631 does not have any role in antiviral activity. In contrast, Mu Mx1 has been recognised as a potent antiviral protein, in which the Mx-mediated antiviral activity is induced by interacting with the viral nucleoprotein (NP) (Zimmermann et al. 2011). Transgenic expression of Mu Mx1 in chicken DF1 cells resulted in a significant reduction in AIV titers and reduced cytopathic effects, indicating the efficacy of Mu Mx1 against multiple AIV subtypes (Garber et al. 1991; Briggs et al. 2024).

####  TRIM

Tripartite motif-containing (TRIM) proteins are a class of E3 ubiquitin ligases that regulate many cellular processes including IFN-induced antiviral processes. The antiviral activity of TRIM proteins is predominantly mediated by either modulating host innate immunity or by direct targeting viral effectors via the ubiquitin-proteasome pathway, wherein viral proteins act as substrates (reviewed in Ozato et al. 2008). TRIM genes are highly conserved across metazoans and belong to the family of Really Interesting New Gene (RING). The domain architecture of TRIM proteins comprises of four principal components: a RING domain, one or two B-box domains, a coiled-coil domain at the amino-terminal region, and a distinct C-terminal domain. Despite the evolutionary diversification and extensive variations of the domain architecture of TRIM proteins, the core function of TRIM proteins in mediating ubiquitination remains conserved across species e.g., for TRIM proteins of human and chicken origin. TRIM-mediated antiviral activity can occur through both IFN-dependent and -independent pathways at multiple stages of the viral life cycle, primarily mediated through K48- or K63-linked polyubiquitination of target viral proteins. For example, IFN-inducible TRIMs, such as TRIM14, TRIM22, TRIM21, TRIM35, and other TRIM proteins facilitate viral restriction by degrading influenza viral proteins, including NP, NS (Non-structural), and PB1 (Polymerase basic 1), in a ubiquitin ligase-dependent manner in human cells (Sun et al. 2020; Husain 2024). Furthermore, knockout of TRIM35 in mice led to an increased susceptibility to influenza infection, highlighting the role that TRIM proteins play in coordinating innate immune response. Fu et al. (2015) demonstrated that TRIM32 detects IAV infection by interacting with the viral PB1 protein, and mediates ubiquitination of PB1 to provide cell intrinsic restriction against IAV infection in human cells. Furthermore TRIM32-mediated restriction was observed across multiple IAV strains in human cells, suggesting that TRIM32 exhibits a broad antiviral mechanism and that viral PB1 has not evolved the ability to evade TRIM32-associated antiviral activity. Similarly, Nenasheva et al. (2021) demonstrated that the transgenic expression of human TRIM14 in mice have resulted in IFN-independent antiviral activity and exhibited a potent decrease in viral replication. Additionally, IFN-encoding genes exhibited lower levels of expression in challenged transgenic mice compared to that of control mice. This study provides a foundation for considering TRIM14 as a potential antiviral transgene to enhance AIV resistance in chickens for broad spectrum antiviral activity.

#### BTN3A3

The butyrophilin (BTN) gene family, located within the mammalian MHC class I region, encodes type I transmembrane proteins characterized by two extracellular immunoglobulin (Ig)-like domains and a cytoplasmic PRYSPRY domain. In humans, three BTN subfamilies have been identified: BTN1, BTN2, and BTN3. The BTN3A subfamily including BTN3A1, BTN3A2, and BTN3A3 arose through tandem duplication and shares ~ 95% sequence identity in their extracellular domains (reviewed in Chen et al. 2021; Kone et al. 2022). BTN3A proteins are broadly expressed in epithelial and immune cells, where they play roles in modulating immune responses. Among the numerous ISGs upregulated during viral infection, BTN3A3 has emerged as a potent, lineage-specific restriction factor against AIVs. Identified through an arrayed ISG overexpression screen in human and macaque cells, BTN3A3 and to a lesser extent its paralogue BTN3A1 markedly suppressed replication of a mallard-origin AIV in human lung epithelial (A549) cells. Overexpression of BTN3A3 alone reduced viral titres by up to 100,000-fold, whereas BTN3A2, which lacks the antiviral PRYSPRY domain, had no effect (Pinto et al. 2023). The restriction is specific to avian viruses. Knockdown of endogenous BTN3A3 in primary human bronchial epithelial cells and fetal lung fibroblasts significantly enhanced replication of the avian virus but had no impact on the human-adapted strain A/Puerto Rico/8/1934(H1N1). Mechanistically, BTN3A3 acts early in infection after viral entry but before robust viral RNA replication impairing accumulation of vRNP complexes in both the cytoplasm and nucleus and triggering IFN responses. However, the precise molecular target and mechanism remain unresolved. Sensitivity to BTN3A3-mediated restriction is governed by the viral NP, particularly residue 313. Avian IAVs typically encode a conserved phenylalanine (313 F) at this site, conferring sensitivity, while human-adapted strains almost exclusively carry resistance-associated tyrosine (313Y) or valine (313 V). This substitution is believed to be a critical step in zoonotic adaptation. Nevertheless, this restriction barrier is not absolute. Although several zoonotic AIVs, such as H7N9, retain the BTN3A3-sensitive 313 F residue, they can still infect humans, indicating that additional viral determinants modulate this phenotype. Pinto et al. (2023) demonstrated that substitution of asparagine at position 52 with tyrosine (52Y) in H7N9 NP restores sensitivity to BTN3A3, implicating residue 52 as a modulatory site. These results suggest that compensatory interactions between residues 52 and 313 can modulate restriction sensitivity and facilitate evasion of BTN3A3-mediated antiviral activity. Together, these findings position BTN3A3 as a key ISG in species-specific restriction of AIVs and highlight NP residues 313 and 52 as molecular determinants of host range and zoonotic potential.

#### RIG-I

Another ISG, RIG-I is one of the primary cytosolic RNA sensors with an emerging role in the nucleus (reviewed in Rehwinkel et al. 2020). It is involved in the spatiotemporal sensing of IAV replication, leading to the induction of type I IFNs. Upon activation, RIG-I undergoes conformational changes and signals via the mitochondrial antiviral signaling protein MAVS, initiating downstream cascades that induce type I and III IFNs and ISGs. This pathway is tightly regulated by ubiquitination, particularly through the E3 ligases TRIM25 and RNF135 (also known as Riplet), which enhance RIG-I activation via K63-linked ubiquitin chains. Unlike waterfowl, chickens naturally lack functional RIG-I and RNF135, a deficiency linked to their increased susceptibility to AIV. Previous studies, such as that by Barber et al. (2010) demonstrated that overexpression of duck RIG-I in chicken DF-1 cells significantly reduced H5 virus replication, indicating that the RIG-I pathway is functional and antiviral when reconstituted in chickens. Building on this, Sid et al. (2023) generated transgenic chickens expressing duck RIG-I alone or together with RNF135 under duck-specific promoters to assess their impact on immune function and AIV susceptibility. Unexpectedly, embryonated eggs and chicken embryo fibroblasts (CEFs) from these transgenics showed no significant difference in replication of low pathogenic AIV (H9N2) compared to wild-type controls, underscoring the importance of promoter context and cell-type-specific regulation. In the uninfected transgenic birds, RIG-I expression alone markedly altered adaptive immune cell populations. These chickens exhibited elevated numbers of αβ and γδ T cells, B cells, and specifically CD4⁺ and CD8α⁻ T cells, with a concurrent decrease in CD8α⁺^high T cells, indicating a role for RIG-I in shaping T cell composition and priming adaptive immunity. In contrast, RNF135 expression alone had no significant effect on immune cell profiles, while co-expression of RIG-I and RNF135 largely normalized immune parameters to wild-type levels suggesting a modulatory role for RNF135 in tempering RIG-I-driven immune activation. Following infection with highly pathogenic H7N1 AIV, clinical outcomes diverged markedly across transgenic lines. RIG-I-expressing birds experienced severe disease, with morbidity reaching 67% within two days post-infection, accompanied by substantial weight loss, elevated viral loads, and widespread tissue pathology. In contrast, co-expression of RNF135 markedly attenuated disease: birds showed reduced viral genome copies in the caecum and lungs, along with milder clinical signs, indicating that RNF135 is essential for modulating RIG-I activity to enable effective viral control without triggering excessive inflammation. RNF135-only birds displayed disease severity and viral replication similar to wild-type controls, confirming that RNF135 is not sufficient to confer protection in the absence of RIG-I. These findings underscore the dual role of RIG-I in antiviral defence and immune modulation, and highlight the necessity of RNF135 for balanced, protective immunity. The evolutionary loss of RIG-I and RNF135 in chickens may represent a trade-off to minimize immunopathology, but at the cost of heightened susceptibility to viral infection (Sid et al. 2023).

## Viral restriction factors identified through high-throughput screening

The traditional methodology for investigating the functional roles of genes predominantly relies on generating cell lines with targeted gene knockouts or overexpression of individual genes. However, the low-throughput nature of these studies often leads to a biased understanding of genes and to the omission of high-confidence targets. In contrast, genome-wide screening facilitates systematic exploration of biological processes on a genome-wide scale under selection pressures, such as viral infections.

### High-throughput screening methodologies

Genome-wide functional screens using RNA interference (RNAi) and CRISPR/Cas9 are powerful tools for systematically identifying host factors that influence immune responses, viral replication, and disease susceptibility. Both RNAi and CRISPR technologies have been widely employed to uncover host determinants crucial for influenza replication. However, both platforms have distinct advantages and trade-offs. For example, RNAi screens are often constrained by incomplete gene silencing and prone to off-target effects, which often leads to heterogeneity of gene silencing within the cell population. In contrast, CRISPR-based knockout screens offer a high degree of consistency in targeting efficiency through gene editing at the DNA level, resulting in complete loss of function. Nevertheless, the effectiveness of CRISPR/Cas9 is often sequence context, albeit the concern of off-targets is considerably lower than that of RNAi. However, it is worth noting that the complete knockout of essential genes using CRISPR/Cas9 could lead to cellular lethality, thereby complicating the interpretation of functional outcomes. Therefore, the choice between the two technologies depends on the specific objectives and context of the genome-wide screen to be performed (reviewed in Housden and Perrimon 2016).

### Steps involved in conducting genome-wide screening

CRISPR-based high-throughput methodologies exploit the versatility of Cas proteins, wherein the expression of a single protein is sufficient, while a library of guide RNAs (gRNAs), each with 20-nucleotide specificity, can be used to target genome-wide. The in-silico component of a pooled library involves the design of 3–4 gRNAs per gene (i.e., approximately 60,000 gRNA targeting 20,000 genes), which are subsequently cloned into a lentiviral CRISPR plasmid. Lentivirus encoding Cas9 and individual gRNA will be transduced into continuous cell lines at a low multiplicity of infection (MOI) to ensure that each individual cell receives a unique gRNA. This is followed by subsequently conducting a deep sequencing to evaluate the coverage, diversity, and representation of the genomically integrated gRNAs within the cell libraries (Han et al. 2018).

Genome-wide screens are carried out using two main approaches: a survival screen and a sort-based screen. In the survival screen, the validated cell library is infected with the virus of interest at a high MOI, followed by successive rounds of harvesting and expansion of surviving cells. By performing multiple rounds of infection on surviving cells, virus resistant cells are progressively enriched by eliminating the susceptible cells in each infection round. These successive rounds of infection are expected to facilitate internal competition among genes by creating a population bottleneck that favours the survival of cells with enhanced viral resilience. On the other hand, the sort-based screen involves the use of flow cytometry to separate phenotypically distinct cells, in which the infected cell library undergoes antibody staining for viral protein prior to analysis. The stained cells are separated for resistant (low virus) and susceptible (high virus) based on the intensity of the viral protein marker. In both screening approaches, amplicon sequencing is conducted on genomic DNA of cells to determine by the relative abundance of specific gRNA sequences, which is then used to identify and rank genes involved in the phenotype of interest (Joung et al. 2017; Han et al. 2018; Li et al. 2020).

### Influenza viral restriction factors identified in CRISPR-based screens

Genome-wide CRISPR screening studies to identify host factors essential for cancer and pluripotent stem cells was first reported by Shalem et al. (2014). By 2018, this approach was extended to the investigation of host-pathogen interactions of influenza virus in human cells. The availability of high-quality reference genomes of livestock species, including chickens, has provided a valuable resource for conducting genome-wide screens. Compared with the extensive application in cell lines of model species, genome-wide functional screens in livestock species have lagged considerably, limiting the potential for rapidly identifying host targets. For example, genome-wide human libraries of KO and activation screens have been extensively used for functional genomic studies in human and mouse cells. These studies have identified numerous host factors, albeit the targets identified in each study have been influenced by the type of screening methodology, cell type used, and virus under investigation. Despite this, host factors identified in these studies have provided a valuable source for the investigation of key orthologous genes in chicken cells, given the evolutionary conservation of antiviral factors between humans, mice, and avian species. Such studies will deepen our understanding of virus-host interactions, reveal targets for precision selection in pure lines, and inform the development of host-directed vaccines and treatments to combat avian influenza in poultry populations.

Genome-wide screening to identify IAV host factors was first conducted in human lung epithelial GeCKO cells using a genome-wide KO library that target approximately 19,050 genes with 65,383 guide RNAs (Han et al. 2018). Using reverse genetics derived low-pathogenic version of H5N1 (VN04Low), the screening methodology implemented a survival screen by performing five rounds of infection of GeCKO cells. This screen has revealed several host factors that are involved in viral entry and cell-intrinsic immune regulation, with a strong enrichment for genes linked to sialic acid biosynthesis and glycosylation. Notably, cells lacking SLC35A1, which is required for the expression of sialylated receptors recognized by influenza viruses, were significantly enriched after H5N1 selection indicating impaired HA binding and viral entry. In another study, Li et al. (2020) conducted a similar genome-wide screen using A549-Cas9 cells infected with the A/Puerto Rico/8/1934 (H1N1) strain. This screen identified three key host factors essential for viral entry and regulation: WDR7, CCDC115, and TMEM199. Additionally, the study highlighted CMTR1, a 2′O-ribose cap methyltransferase, as a critical factor for the cap-snatching mechanism employed by the virus. Beyond its role in viral RNA processing, CMTR1 was also identified as a key regulator of cell-intrinsic immune surveillance during IAV infection. In a separate study, a knockout screen using H1N1 and A549 cells uncovered the role of GNE (glucosamine [UDP-N-acetyl]−2-epimerase/N-acetylmannosamine kinase) in supporting IAV replication (Ma et al. 2023). The loss of GNE expression in host cells significantly impaired replication across multiple IAV subtypes, while its reintroduction partially restored infection. Conversely, GNE overexpression enhanced IAV replication in wild-type cells. Mechanistically, GNE knockout reduced the expression of both α−2,3- and α−2,6-linked sialic acids, thereby hindering viral adsorption and endocytosis.

Munir et al. (2024) performed a genome-wide CRISPR activation screen in HeLa cells infected with the A/Puerto Rico/8/1934 (H1N1) strain and identified previously uncharacterized antiviral genes. Among them, JADE3 (Jade family PHD zinc finger 3) is critical for restricting influenza virus replication. JADE3 recruits histone acetyltransferases to the ORC1 complex, modulating chromatin to regulate transcription. It is required for both constitutive and inducible expression of IFITM3 (IFN-induced transmembrane protein 3), acting through activation of the NF-κB signalling pathway. These findings position JADE3 as a key regulator of an NF-κB-dependent antiviral program that promotes IFITM3 expression and limits IAV infection. Similarly, Heaton et al. (2017) conducted a systematic screening of A549 cells to identify host factors whose overexpression could restrict A/Puerto Rico/8/1934 (H1N1) viral infection. The major hit from their screen, B4GALNT2, demonstrated inhibitory activity against influenza viruses, with a preference for α2,3-linked sialic acid receptors. Overexpression of B4GALNT2 blocked infection by all tested AIV strains, including the H5, H9, and H7 subtypes which have been previously associated with human disease. In a subsequent study by the same group, a CRISPR activation screen using A549 cells infected with influenza B virus (IBV) identified beta-1,3-glucuronyltransferase 1 (B3GAT1) as a potent host factor that restricts viral entry. B3GAT1 was also effective against H1N1 and H3N2 IAV strains, both of which rely on sialic acid–mediated cell entry. Notably, expression of B3GAT1 in primary human respiratory epithelial cultures and in mouse models significantly limited influenza pathogenesis, supporting its potential as a host-directed, broad-spectrum antiviral target (Trimarco et al. 2022). More recently, Jin et al. (2025) conducted the first genome-wide CRISPR knockout screen in chicken cells, uncovering previously unrecognised host factors that facilitate AIV replication. Key candidates identified include RNF2, a negative regulator of ISG expression; DCP1A, associated with transcriptional networks that promote viral replication, and CREB3L3, a transcription factor potentially involved in cholesterol regulation critical for viral entry.

Although numerous host restriction factors have been identified in vitro, relatively few have been validated in vivo. For example, Mx1 has demonstrated potent antiviral activity both in vitro and in vivo; in challenge studies, Mx1-expressing mice were protected from lethal HPAI infection, whereas Mx1-deficient mice succumbed (Tumpey et al. 2007). BTN3A3, identified as a potent restriction factor in human cells and shown to inhibit AIV replication in vitro, is a strong candidate for in vivo validation in chickens. Its co-expression with Mx1 may provide synergistic effect against infection, although this remains to be experimentally validated. B4GALNT2 modifies α2,3-linked sialic acid–containing glycans and has been shown to significantly reduce AIV susceptibility in vitro; its transgenic expression in chicken fibroblast cells restricted replication of multiple AIV strains, including H5N8 and H9N2 (Park et al. 2023), making it a compelling candidate for in vivo testing. In contrast, SLC35A1, a sialic acid transporter essential for glycan-mediated viral entry and ranked among the top host-dependency factors in several studies plays critical roles in fundamental cellular processes, rendering it an impractical target for gene knockout in chickens. Endothelial-specific deletion of SLC35A1 in mice led to the loss of liver sinusoidal endothelial cell (LSEC) identity and increased neonatal lipid accumulation, suggesting risks of embryonic lethality or developmental abnormalities (Zuo et al. 2024). Ultimately, it is important that these in vitro findings must be validated in vivo to determine their potential for virus antagonism and reducing viral transmission in poultry, as well as their effects on host development, immune regulation, and animal welfare.

## Targeting the influenza genome: RNA-based antiviral platforms

Antiviral strategies that directly target viral genomes are emerging as highly specific and robust modalities. The promise of nucleic acid-based targeting is particularly amenable to influenza because of its relatively compact genome size and dependency on a set of essential genes for replication, including those encoding RNA-dependent RNA polymerase subunits (PB1, PB2, and PA) and NP, all of which are crucial for transcription, replication, and genome packaging. Among the several characterized tools, RNA interference (RNAi) and CRISPR-Cas13 are the two most promising nucleic-acid platforms for mediating sequence-specific RNA degradation (Novina and Sharp 2004; Cox et al. 2017). RNAi harnesses the highly conserved endogenous RNA-silencing pathway by 21-nucleotide small interfering RNAs (siRNAs), whereas CRISPR-Cas13 uses 28–30 nucleotide CRISPR RNAs (crRNAs) to guide the Cas13 ribonuclease. Unlike protein-based strategies (e.g., scFvs or synthetic effectors) (Li et al. 2016), RNAi and Cas13 technologies bypass the need for extensive discovery pipelines and are often rapid, precise, and versatile in designing antiviral effectors. Moreover, these technologies offer broad targeting flexibility, especially against conserved genomic regions critical for viral replication.

The use of RNAi against AIV was first demonstrated by delivering siRNAs into chicken embryonic cells and embryonated eggs. The siRNAs targeting conserved regions of AIV have effectively suppressed replication across multiple influenza subtypes. Targeting NP and PA genes of influenza with siRNAs led to a reduction in mRNA levels while also inhibited the synthesis of cRNA and vRNA, suggesting the siRNA mediated interference suppressed viral replication cycle. Importantly, the inhibition was dependent on the presence of a functional antisense strand in the siRNA duplex, indicating that viral mRNA was the primary target (Ge et al. 2003). In a similar study by Piasecka et al. (2020), siRNAs designed to target the conserved motifs of NP mRNA, which forms an RNA secondary structure, resulted in marked reduction of IAV replication in MDCK cells, underscoring the potential of RNA structure-guided siRNA design in enhancing antiviral potency. In parallel, CRISPR-Cas13 systems have been harnessed for the sequence-specific targeting of influenza viruses. Freije et al. (2019) repurposed Cas13a and Cas13b systems for IAV targeting in MDCK and human A549 cells by demonstrating RNA degradation and suppression of viral replication. Similarly, Challagulla et al. (2021) reported CRISPR-Cas13a mediated targeting of influenza virus in avian cells by generating stable chicken DF1 cells expressing Cas13a. In this study, a multiplexed crRNA vector targeting the PB1, NP, and M gene segments transfected into Cas13a stable cells resulted in significantly reduced viral titers of A/WSN/1933 (H1N1) and A/Puerto Rico/8/1934 (H1N1). Although CRISPR/Cas13 systems were shown to mediate robust and sequence-specific RNA targeting, the characteristic collateral activity of these nucleases remains a significant safety concern. This collateral degradation of bystander RNAs can lead to unintended cellular effects and potentially constrain its use in vivo.

Recent breakthroughs in de novo protein design that leverage artificial intelligence are facilitating the design of bespoke antiviral proteins with high specificity and defined functional properties. De novo protein design was first employed for the development of functional binders targeting the conserved stalk region of influenza HA by David Baker’s research group, using the Rosetta protein design platform (Fleishman et al. 2011). One of the striking features of de novo designed proteins is their exceptional compactness and flexibility to design antivirals at the interface of highly conserved or functionally critical regions of viral epitopes that exhibit low mutation rates. In contrast to conventional antibodies, which are produced as part of a trial-and-error approach by the host immune response, de novo protein design enables targeted and iterative optimization to improve the binding affinity and protective efficacy. Given that de novo proteins can be genetically encoded for intracellular expression, the possibility of transgenic expression remains unexplored (Chevalier et al. 2017; Quijano-Rubio et al. 2021). In particular, potential immune responses, such as the development of neutralizing antibodies or hypersensitivity reactions against de novo proteins, must be carefully considered.

## Genetic engineering in chickens: from viral vectors to precision editing

Genetic engineering technologies that enable targeted gene knockouts and transgene overexpression in vivo have revolutionized functional genomics in animal agriculture, including avian species. These advancements have provided powerful tools for elucidating gene function, modelling diseases, and developing traits of agricultural and biomedical relevance. The early efforts of generating transgenic avian models have employed retroviral vectors such as avian leukosis virus (Salter and Crittenden 1989) and reticuloendotheliosis virus (Bosselman et al. 1989) to transduce blastodiscs of developing embryos. However, the use of viral vectors is often faced with challenges such as transgene silencing, cargo size restrictions, and low germline transmission efficiency. Subsequent studies employing lentiviral vectors have shown improved germline transmission rates, albeit with the challenge of random genomic integration (McGrew et al. 2004).

**Fig. 2:**
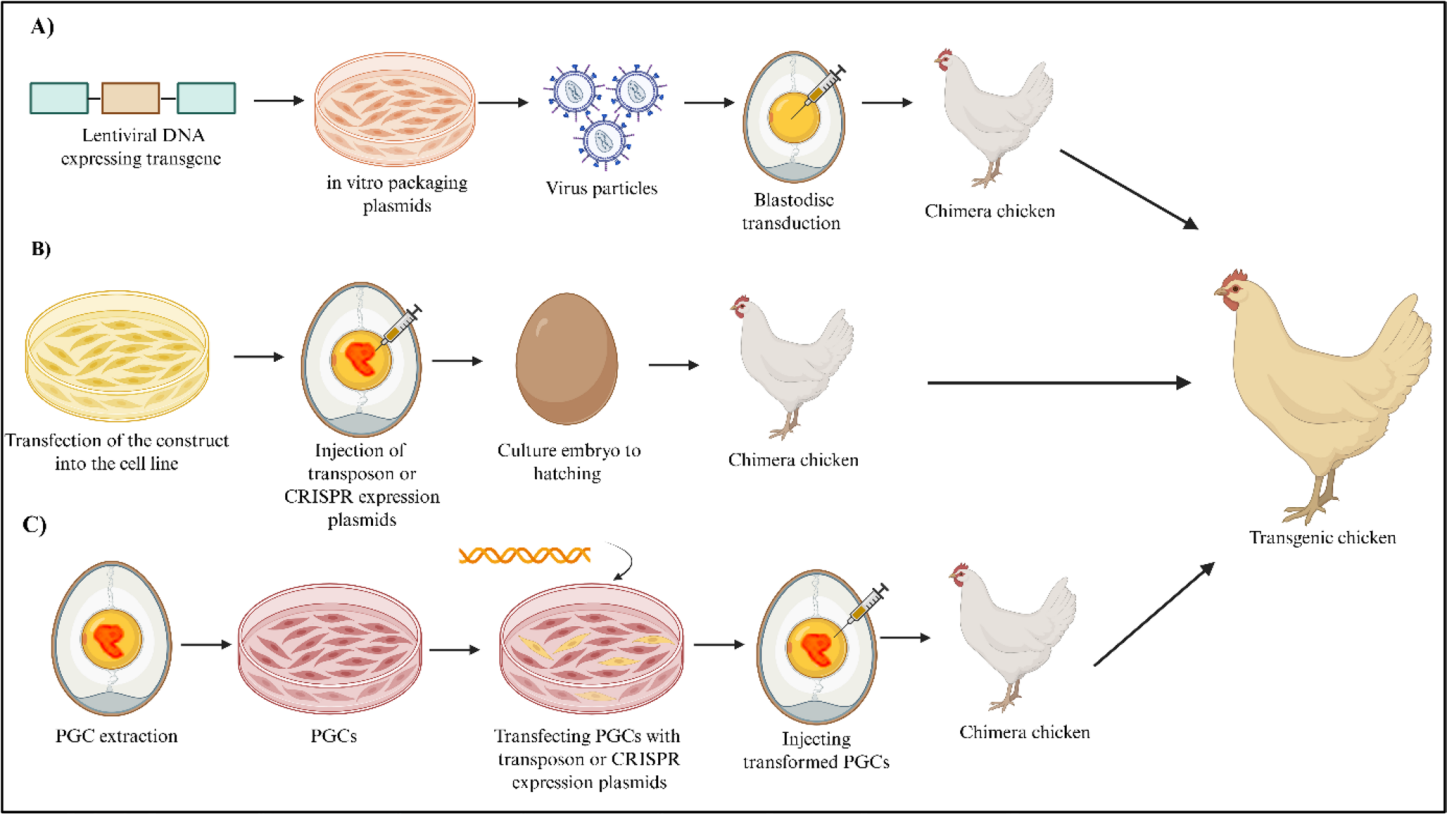
Methods for Generating Transgenic Chickens (**A**) Targeting primordial germ cells (PGCs) with retroviral vectors. (**B**) Direct transfection of PGCs in ovo. (**C**) In vitro transfection of PGCs. Following these methods, the process involves embryo development, chimera formation, crossing chimeras with wild-type chickens, and the generation of transgenic offspring. Created with BioRender.com. Adapted from (Fallahi and Mohammadhassan 2020; Petitte and Mozdziak 2007)

A major breakthrough in avian genetic engineering was the establishment of protocols for the isolation and long-term culture of avian primordial germ cells (PGCs) (van de Lavoir et al. 2006; Lee et al. 2016; Taylor et al. 2017). PGCs are embryonic precursor cells for sperm and ova, and their accessibility for in vitro modification has made germline modification in chickens efficient and reliable. Transfection of gene-targeting vectors into cultured PGCs enables the generation of clonal PGC populations carrying the desired genetic modification. These genetically modified PGCs can then be injected into HH stage 14–15 recipient embryos to produce germline chimeras. Subsequent breeding of chimeras will result in germline-edited offspring carrying the desired knockout or transgene. Early applications of PGC-based transgenesis involved the use of non-viral gene delivery systems, such as the Tol2 transposon and PiggyBac transposition systems. These systems have been shown to mediate transgenes up to 10 Kb in size, facilitating efficient and stable genomic insertion of transgenes of interest into avian genomes (Macdonald et al. 2012; Park and Han 2012). Furthermore, the use of Tol2 transposon vectors targeting circulating PGCs in Hamburger-Hamilton (HH) stage 14–15 embryos has been shown to mediate the germline integration of transgenes (Tyack et al. 2013) (Fig. 2). A major advantage of in vivo transfection approach is that genetic modification can be achieved without establishing and maintaining germ cell culture. By simply injecting the plasmids such as Tol2 transposon vectors into early-stage embryos, this approach enables a practical platform for germline engineering possible in avian species lacking optimised PGC culture conditions (Serralbo et al. 2020). A recent study also reported the generation of immune receptor knockout chickens via direct in vivo transfection of primordial germ cells (Jenkins et al. 2025).

Building on these advancements, subsequent studies progressed from random transgene integration to the generation of targeted gene knockout chicken models. Schusser et al. (2013) reported the generation of the first gene-knockout chicken line by targeting the joining (J) gene segment of the chicken Ig heavy chain gene by homologous recombination in cultured PGCs to generate a B cell-less chicken. The targeting strategy involved the use of a recombination vector carrying homology arms of approximately 8 kilobases in length, followed by transfection into cultured PGCs. Despite successfully achieving the gene-targeting, the isolation of correctly targeted PGC clones required the transfection of at least 10⁷ cells, highlighting the need for strategies to improve targeting outcomes.

The advent of programmable nucleases such as ZFNs, TALENs, and CRISPR/Cas9 have revolutionized avian genome engineering by enabling the generation of gene-edited and transgenic avian models at high-efficiency (Gaj et al. 2016; Sid and Schusser 2018). Compared to the classic homologous recombination, programmable nucleases dramatically enhance gene targeting by introducing double strand breaks at a predefined genomic locus. Among them, CRISPR/Cas9 has become the widely adopted system for chicken genome editing, facilitating the creation of several gene-edited models (reviewed in Khwatenge and Nahashon 2021). Given the focus of this review is on gene-edited chickens for disease resilience, many of the landmark studies in developing gene editing in chicken were not discussed but were covered in the following review articles (Park et al. 2020; Ledesma and Van Eenennaam 2024; Gallala 2025). Recently, Ballantyne et al. (2021) developed an inducible sterile surrogate host chicken line with an aim to accelerate the development of gene edited models in a single generation. In this model, endogenous germ cells of both male and female birds are selectively ablated to exclusively support the development of exogenous donor-derived PGCs, enabling the development of genetically modified lines at high efficiencies. Collectively, these innovations have substantially accelerated progress in avian functional genomics and hold considerable promise for future poultry breeding and health enhancement applications. The following sections will highlight key studies that made progress in improving avian influenza resilience in chickens.

### Genetic modification for disease resistance or resilience in chicken

Genetic modification to confer avian influenza resistance in chickens was first demonstrated by stable insertion of a synthetic RNA hairpin into the chicken germline. In this study, a D5 decoy molecule that encompasses conserved influenza genomic termini from both the 3′ and 5′ ends was developed. This decoy RNA molecule functions as a competitive substrate for the viral polymerase and sequesters the polymerase complex, resulting in selective inhibition of influenza replication. A lentiviral vector carrying the D5 decoy was used to generate transgenic chickens constitutively expressing the D5 decoy molecule, followed by challenge with H5N1 to assess viral suppression in transgenic chickens. Although both transgenic (TG-D5) and non-transgenic chickens exhibited comparable mortality following direct H5N1 infection, the transgenic chickens exhibited notable reduced viral transmission dynamics. Importantly, wild-type contact birds housed with virus-infected TG-D5 chickens were significantly protected with reduced mortality and limited evidence of viral spread in the wild-type birds. These findings underscore the potential of genetic modification as a population-level intervention to mitigate the spread of highly pathogenic avian influenza at the flock level (Lyall et al. 2011).

To evaluate whether transgenic expression of single-chain variable fragments (scFvs) provides avian influenza resilience, June et al. (2017) generated transgenic birds expressing the 3D8 scFv protein. The 3D8 scFv was originally developed as a tool to induce sequence-independent hydrolytic activity against RNA and DNA, which was later repurposed as a broad-spectrum antiviral approach (Jun et al. 2010; Lee et al. 2014).The transgene encoding 3D8 scFv was introduced into the chicken germline using lentiviral-mediated transgenesis. Transgenic expression of 3D8 scFV did not cause any developmental abnormalities across multiple generations. Transgenic 3D8 chickens that were directly inoculated with LPAI H9N2 seroconverted, similar to the wild-type chickens. However, when transgenic 3D8 chickens were exposed to H9N2 LPAI through in-contact route, reduced levels of viral shedding and minimal immune responses were observed in transgenic chickens compared to that of non-transgenic chickens. These results suggest that transgenic chickens exhibited increased resilience to H9N2 infection. However, the continuous viral shedding observed in transgenic chickens raises concerns about possible emergence of escape mutants, necessitating further studies involving viral evolution under selective scFV pressure.

The use of innate immune effectors as a broad-spectrum antiviral strategy for controlling influenza virus in poultry has been an active area of research. Members of the IFN-induced transmembrane (IFITM) family and IFN-induced proteins with tetratricopeptide repeats (IFITs) have been extensively studied using transgenic chickens. The first study generated a stable chIFIT5 expressing chicken line using an RCAS-based gene delivery strategy (Rohaim et al. 2018). The overexpression of chIFIT5 did not affect the viability of transgenic chickens, albeit the degree of hatchability of the transgenic chicks was compromised. Challenge studies using a sub-lethal dose (EID50 10^5^) of HPAI H5N1 revealed that chIFIT5 expressing chickens have a delayed onset of clinical disease and reduced viral shedding compared to wildtype chickens. However, the transgenic chickens infected with low infectious dose (EID50 10^4^) showed marked resistance against infection compared to wildtype chickens. In a similar study, Rohaim et al. (2021) generated a line of transgenic chickens overexpressing chIFITM1. Unlike chIFIT5 expressing chickens, overexpression of chIFITM1 did not result in any negative effects on embryonic development or hatchability of chickens. The challenge of transgenic chickens expressing chIFITM1 with H5N1 have shown to reduce viral replication and tissue damage in the respiratory tract compared to wild-type chickens. The protection of transgenic chickens from influenza challenge is associated to higher levels of chIFITM1 expression in the trachea. This aligns with the RCAS vector’s tendency to target endothelial cell–rich regions. Together, these two studies provided mechanistic insights into innate immune effectors of host–virus interactions and a foundation for future breeding strategies to enhance resilience against avian influenza.

In a recent study, Idoko-Akoh et al. (2023) developed genome-edited chickens with modifications in ANP32A, a key host cofactor that influenza polymerase exploits for efficient replication in host cells. Comparative genomics studies have revealed that chicken ANP32A (chANP32A) protein contains a unique 33-amino acid insertion, that is absent in mammalian orthologs (Long et al. 2016). Moreover, chANP32A functions as the primary pro-viral factor in chicken cells by supporting the polymerase activity of avian-origin influenza viruses, while chANP32B has a redundant role in supporting polymerase activity. Subsequent domain-swap and systematic mutagenesis studies between chANP32A and chANP32B revealed two critical residues: asparagine at position 129 (N129) and aspartic acid at position 130 (D130), for supporting polymerase activities. Substitution of these residues in chANP32A with their chANP32B counterpart (i.e. N129I and D130N) significantly diminished chANP32A ability to support AIV polymerase function. Importantly, the N129I mutation disrupts a key electrostatic interaction between ANP32A and the viral polymerase complex, resulting in a loss of host-virus compatibility necessary for viral replication (Long et al. 2019). Follow-up studies in chickens were conducted by Idoko-Akoh et al. (2023), in which gene-edited chickens were developed using CRISPR/Cas9- mediated germline genome editing to introduce the N129I and D130N mutations and subsequent assessment of their effect on viral replication in vivo. Although ANP32A protein is implicated in a diversity of physiological processes, targeted editing of the two amino acid residues did not cause any developmental abnormalities. Following exposure to a viral challenge of 10^3^ PFU of the LPAI H9N2-UDL virus, gene-edited birds demonstrated a significant reduction in viral shedding, with no subsequent transmission. However, when subjected to a higher infectious dose of 10^6^ PFU of H9N2-UDL, a proportion of birds exhibited low-level sporadic oral shedding, indicating limited breakthrough infection. Subsequent analysis of the sequence of the virus that was shed from gene-edited chickens revealed that H9N2 had acquired adaptive mutations in the viral polymerase complex. These mutations enabled the virus to better interact with the edited ANP32A protein and other ANP32 family members as cofactors. These findings highlight the dynamic interplay at the virus–host interface, underscoring the plasticity and adaptive potential of influenza viruses in response to host genetic modifications. Although the ANP32A gene edited chicken study is a major advance in using gene editing to enhance influenza resistance in poultry, it also underscores the need for developing alternative strategies that may help completely block viral replication preventing the virus from exploiting any ANP32 family member.

While gene editing and transgenic-based applications have demonstrated the potential to improve disease resilience, factors such as genetic background, breed, and age must be considered, as these factors may influence overall resistance outcomes (Islam et al. 2020). The fact that in vivo proof-of-concept studies did not show any negative effects (Lyall et al. 2011; June et al. 2017; Rohaim et al. 2018; Idoko-Akoh et al. 2023), they provide an encouraging pathway for advancing host-directed genetic strategies for disease resilience in commercial poultry. However, previous efforts to improve disease resistance trait in production animals are often associated with trade-offs affecting other economically important productivity traits (reviewed by Chen et al. 2025). Moreover, variation in the expression of innate immune genes has been shown as a key driver of increased resistance to infection, confirming that the breed and age of chickens play key role influenza susceptibility (Islam et al. 2020). For instance, inbred lines infected with LPAI A/Turkey/England/647/77 (H7N7) display significant variation in viral shedding (Ruiz-Hernandez et al. 2016). Thai indigenous chickens shed less HPAI H5N1 than White Leghorns (Matsuu et al. 2016), and Fayoumis shed less LPAI H5N2 than Leghorns (Wang et al. 2014; Looi et al. 2018). Moreover, the age of birds is another key factor that may have a strong influence on influenza susceptibility, as younger birds typically have less mature immune systems. However, Bertran et al. (2016) reported no measurable age-related differences in broiler susceptibility to H5N2 clade 2.3.4.4 HPAI. Therefore, evaluation of gene-edited and transgenic approaches within genetic background of commercial breeds is essential to determine any potential fitness costs and their practical applicability.

## Conclusions

Since the emergence of H5N1 HPAI two decades ago, a catastrophic panzootic has spread across wild bird populations, becoming a major public health issue with a growing frequency of spillover to mammals, including humans. From a poultry health perspective, HPAI continues to devastate the poultry industry globally, and there is an urgent need for science-driven, collaborative solutions that integrate innovative strategies to mitigate its impact. Central to these efforts will be the development of host-directed approaches for improving resilience of poultry populations, in combination with vaccination efforts and biosecurity measures. In this context, emerging technologies such as the genome-wide screens and single-cell RNA sequencing (scRNA-seq) are offering an unprecedented opportunity to uncover novel genetic variants linked to disease resistance. For example, the Chicken Genotype-Tissue Expression (ChickenGTEx) project represents a major step in empowering our understanding of the regulatory architecture underlying complex phenotypes, such as disease resistance or resilience. This study provides a pilot reference of regulatory variants across transcriptomes from 28 chicken tissues spanning protein-coding genes, long non-coding RNAs (lncRNAs), and exons, as well as on post-transcriptional processes such as alternative splicing and 3′ untranslated region (3′UTR) alternative polyadenylation (Guan et al. 2025). The delineation of such a complex regulatory framework in resilience traits has the potential to identify critical genetic variants for disease-resistant phenotypes. Nevertheless, decoding these variants will require multidisciplinary efforts to identify high-confidence disease targets for gene-editing.

As the application of gene-editing technologies continue to progress, it is critical to address the risk of viral escape through adaptive mutations, impact of intended editing on key production traits such as egg laying performance, body weight, and overall fitness to ensure commercial viability and animal welfare are not compromised. Furthermore, public perception regarding the use of GM in the food chain must be proactively addressed through rigorous safety assessments, transparent communication and broader public and stakeholder engagement. In conclusion, as the threat of HPAI continues to escalate, the integration of innovation techniques for high-confidence target discovery, together with advances in gene-editing in poultry could offer sustainable disease management solutions and help reduce the burden of HPAIV on poultry industry.

## Data Availability

No datasets were generated or analysed during the current study.
